# P-1402. Long-term costs and health outcomes of biomarker-based tests to reduce tuberculosis treatment duration in South India: A modeling study

**DOI:** 10.1093/ofid/ofaf695.1589

**Published:** 2026-01-11

**Authors:** Palak Shah, Madolyn Dauphinais, Pranay Sinha

**Affiliations:** Boston University Chobanian and Avedisian School of Medicine, Boston, Massachusetts; Boson Medical Center, Boston, Massachusetts; Boston University, Boston, Massachusetts

## Abstract

**Background:**

While effective, tuberculosis (TB) treatment is long and burdensome. Biomarker-guided strategies to shorten therapy have the potential to reduce costs and improve adherence, but the conditions under which such strategies are cost-effective remain unclear.

**Table 1.**
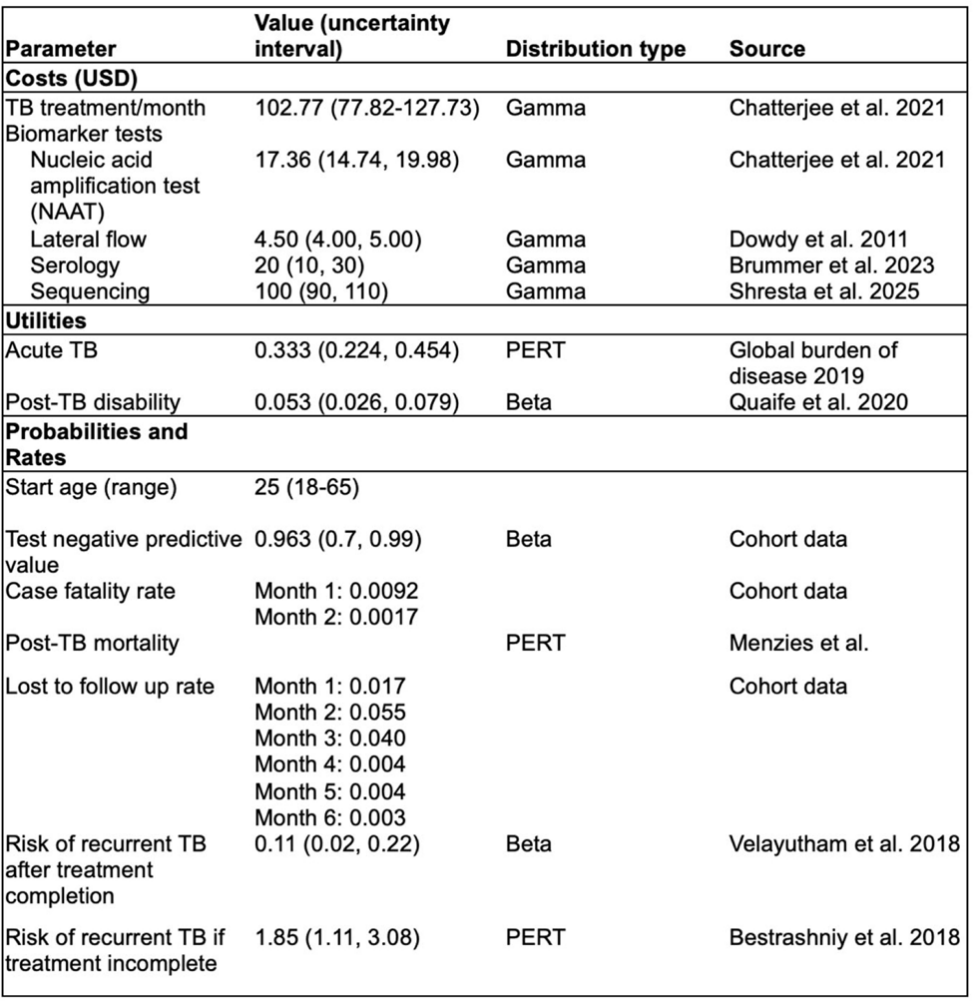
Key model inputs and assumptions

**Table 2.**
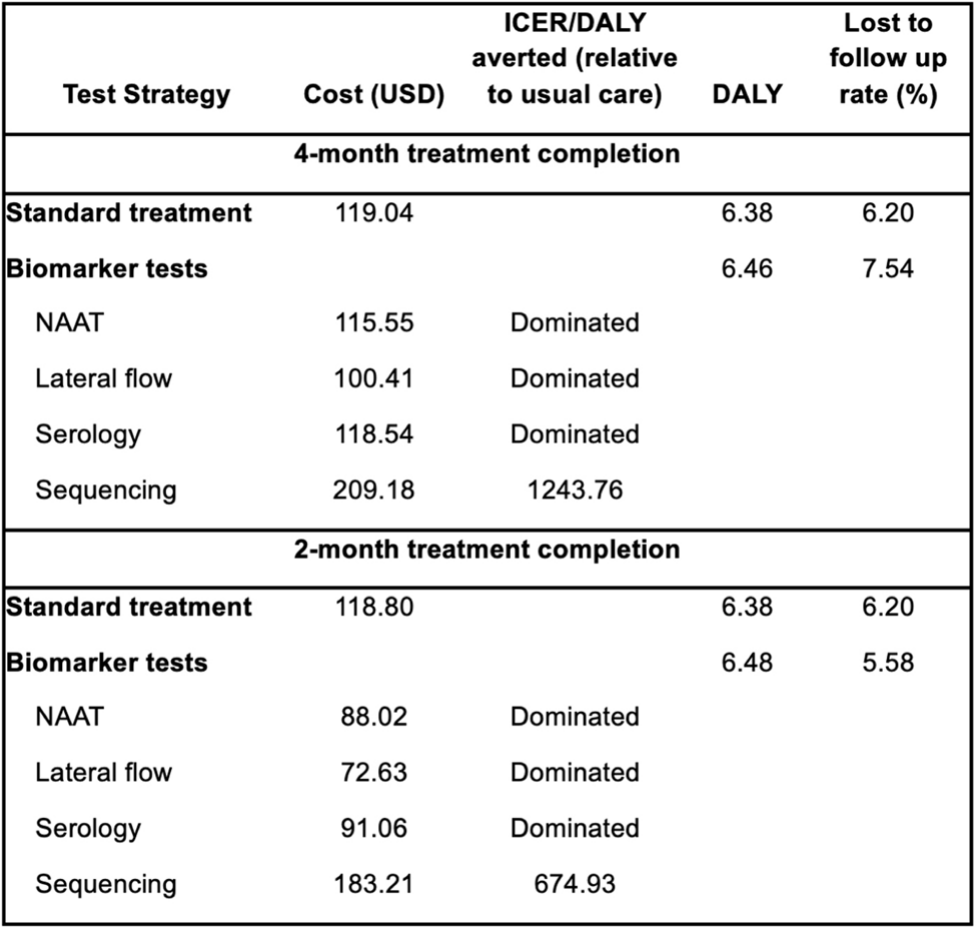
Model outputs

**Methods:**

We developed a Markov model to evaluate the cost-effectiveness of biomarker-guided treatment shortening in South India. Three scenarios were modeled: standard six-month therapy and biomarker-guided completion at four or two months. Outcomes included costs, disability-adjusted life years (DALYs), and incremental cost-effectiveness ratios (ICERs). We conducted one- and two-way sensitivity analyses varying test cost and negative predictive value (NPV), and generated cost-effectiveness acceptability curves (CEACs).

**Figure:**
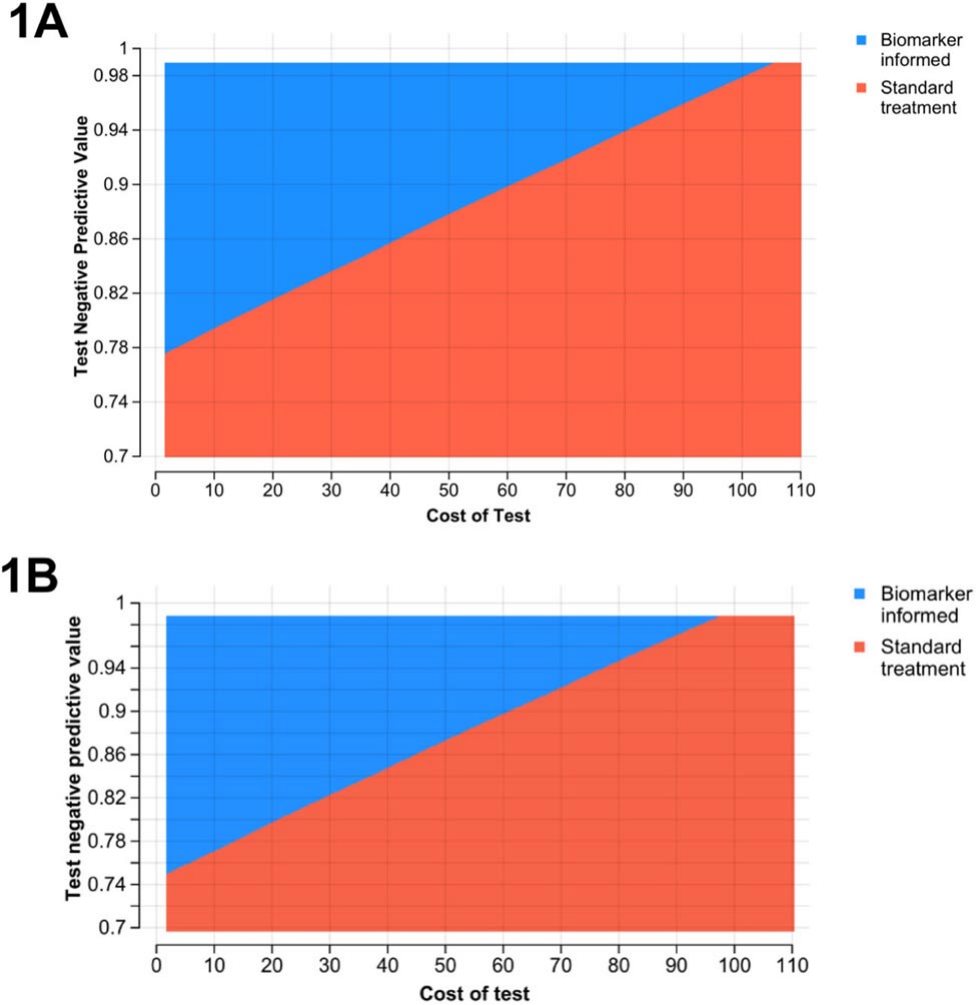
Two-way sensitivity analysis of test cost and negative predictive value 1A) Analysis with treatment completion at two months at $571.50 willingness to pay threshold; 1B) Analysis with treatment completion at four months at $571.50 willingness to pay threshold

**Figure:**
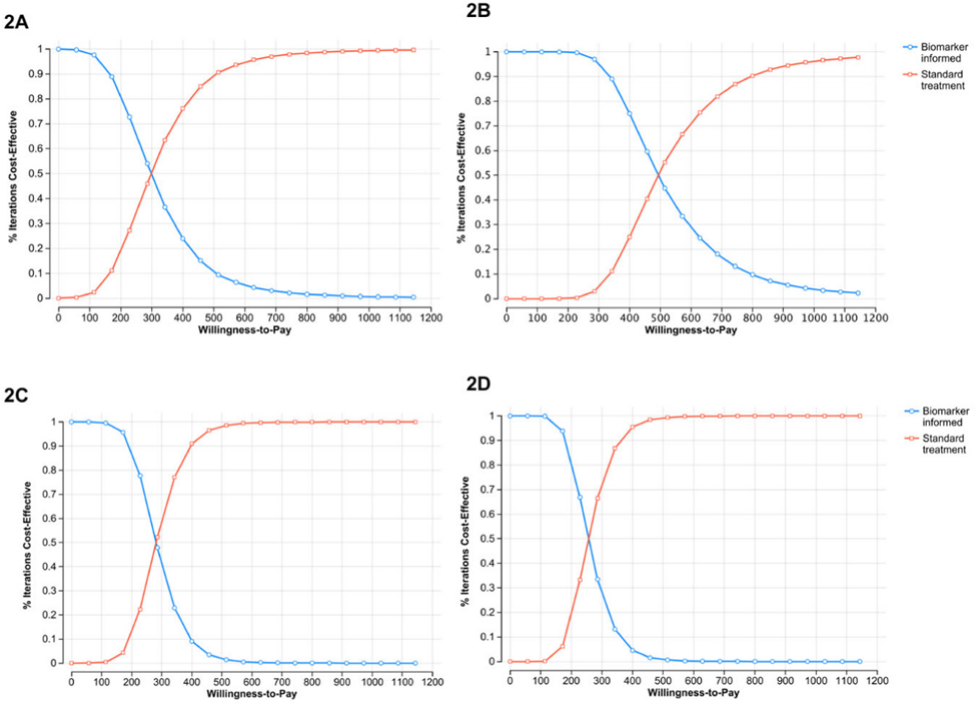
Cost-effectiveness acceptability curves for biomarker testing relative to standard therapy. 2A) Serology testing at two months; 2B) lateral flow testing at two months; 2C) Serology testing at four months; 2D) lateral flow testing at four months

**Results:**

At a willingness-to-pay threshold of $571.50 per DALY averted, biomarker-guided treatment at four or two months was cost-saving compared to standard therapy for all test types except sequencing. For two-month strategies, ICERs ranged from dominance (nucleic-acid amplification test, lateral flow, and serology) to $675/DALY averted (sequencing). At four months, all non-sequencing tests dominated standard care. In one-way sensitivity analysis, results were most sensitive to test cost and NPV. Two-way analysis revealed that two-month strategies were cost-effective if NPV≥0.77 and test cost ≤$105; four-month strategies were cost-effective if NPV ≥0.75 and test cost ≤$96. CEACs showed serology and lateral flow tests had >80% probability of being cost-effective across most simulated scenarios at both time points.

**Conclusion:**

Biomarker-guided treatment shortening can be highly cost-effective in high-burden settings, particularly if test costs are low and NPVs high. These findings can inform target product profiles for TB biomarkers.

**Disclosures:**

All Authors: No reported disclosures

